# COVID-19 Vaccine Acceptability and Financial Incentives among Unhoused People in Los Angeles County: a Three-Stage Field Survey

**DOI:** 10.1007/s11524-022-00659-x

**Published:** 2022-05-31

**Authors:** Allison D. Rosen, Jacqueline Beltran, Emily Thomas, Jonni Miller, Brooke Robie, Savanah Walseth, Shea Backes, Nicolas Leachman, Alicia H. Chang, Anna Bratcher, Ashley Frederes, Ruby Romero, Ivan Beas, Julissa Alvarado, Brenda Cruz, Michelle Tabajonda, Chelsea L. Shover

**Affiliations:** 1 Housing for Health, Los Angeles County Department of Health Services, Los Angeles, CA USA; 2 Department of Epidemiology, UCLA Fielding School of Public Health, Los Angeles, CA USA; 3 Department of Community Health Sciences, UCLA Fielding School of Public Health, Los Angeles, CA USA; 4 Division of General Internal Medicine and Health Services Research, UCLA David Geffen School of Medicine, Los Angeles, CA USA; 5 Community Field Services, Los Angeles County Department of Public Health, Los Angeles, CA USA; 6 Department of Environmental Health Sciences, UCLA Fielding School of Public Health, Los Angeles, CA USA; 7 Department of Health Policy and Management, UCLA Fielding School of Public Health, Los Angeles, CA USA

**Keywords:** Housing, COVID-19 vaccination, Unhoused people

## Abstract

Unhoused people have higher COVID-19 mortality and lower vaccine uptake than housed community members. Understanding vaccine hesitancy among unhoused people is key for developing programs that address their unique needs. A three-round, rapid, field-based survey was conducted to describe attitudes toward COVID-19 vaccination. Round 1 assessed vaccine brand preference, round 2 assessed intention to accept a financial incentive for vaccination, and round 3 measured vaccine uptake and assessed reasons for vaccine readiness during implementation of a financial incentive program. A total of 5177 individuals were approached at COVID-19 vaccination events for unhoused people in Los Angeles County from May through November 2021. Analyses included 4949 individuals: 3636 (73.5%) unsheltered and 1313 (26.5%) sheltered. Per self-report, 2008 (40.6%) were already vaccinated, 1732 (35%) wanted to get vaccinated, 359 (7.3%) were not yet ready, and 850 (17.2%) did not want to get vaccinated. Brand preference was evenly split among participants (Moderna 31.0%, J&J 35.5%, either 33.5%, *p* = 0.74). Interest in a financial incentive differed between those who were not yet ready and those who did not want to get vaccinated (43.2% vs. 16.2%, *p* < 0.01). After implementing a financial incentive program, 97.4% of participants who indicated interest in vaccination were vaccinated that day; the financial incentive was the most cited reason for vaccine readiness (*n* = 731, 56%). This study demonstrated the utility of an iterative, field-based assessment for program implementation during the rapidly evolving pandemic. Personal engagement, a variety of brand choices, and financial incentives could be important for improving vaccine uptake among unhoused people.

## Introduction

Unhoused people are more likely to die from COVID-19 than housed community members, yet COVID-19 vaccination rates are lower in this population [1, 2]. As of November 2021, fewer than 60% of unhoused people in Los Angeles County are fully vaccinated, compared to 73% of the general population in Los Angeles County [3, 4]. Numerous economic, structural, and racial disparities, both historical and current, underlie this vaccination gap [5]. In order to engage unhoused people in COVID-19 vaccination efforts, further research is needed to better understand reasons for vaccine hesitancy and to inform programs tailored to their unique needs.

Early studies suggest that up to half of unhoused people may be hesitant about getting vaccinated, compared to an estimated 30% of the general population [6, 7]. Common reasons for hesitancy include mistrust of the government as well as concerns about side effects, safety, and long-term health complications [6, 8–10].

One factor that may contribute to vaccine hesitancy among unhoused people is the brand of vaccine being offered. The single-dose Johnson & Johnson COVID-19 vaccine can address key barriers for unhoused people who may be more difficult to find and re-engage for a second dose, but the US pause in administration of the Johnson & Johnson COVID-19 vaccine in April 2021, as well as perceived differences in the effectiveness compared to two dose mRNA vaccines, may impact confidence in this vaccine [11]. While vaccine brand preference has not been considered among unhoused people, a study of American adults found that vaccine brand was not associated with willingness to get vaccinated [12]. However, characteristics that may be related to vaccine brand were associated with willingness to get vaccinated: vaccine efficacy, risk of side effects, FDA approval, cost, and number of doses [12, 13].

Financial incentives may be an important strategy for addressing vaccine hesitancy among unhoused people. To date, there have been conflicting findings on the effects of financial incentives for COVID-19 vaccination in the general population. Early data showed that a statewide lottery in Ohio did not significantly increase vaccination rates [14]. Additionally, a survey of American adults revealed that incentives might discourage COVID-19 vaccine uptake in the USA [12]. However, a pilot incentive program in North Carolina found that that offering $25 gift cards decreased the rate at which people decline vaccination [15].

Financial incentives have been shown to improve vaccine uptake in vulnerable and historically marginalized populations. The North Carolina pilot program reported that participants who had lower income or identified as non-White were more likely to cite the incentive as an important reason for vaccine readiness [15]. Also, a meta-analysis assessing interventions in people who inject drugs demonstrated a sevenfold increase in hepatitis B vaccine completion when compared to no intervention. In this analysis, financial incentives were more effective than all other strategies, including motivational interviewing, intensive case management or peer support, and accelerated vaccine schedules [16]. Finally, qualitative findings from a study of unhoused people found that participants were interested in financial incentives for COVID-19 testing, which may translate to acceptance of incentives for vaccination [9]. However, additional research is needed, particularly among unhoused people, as there is also concern that financial incentives may be viewed as coercive by the very people they are designed to incentivize [17–20].

This study aimed to describe attitudes toward COVID-19 vaccination among unhoused people in Los Angeles County by assessing reasons for readiness and hesitancy, vaccine brand preference, and intention to accept a financial incentive for vaccination as well as by evaluating concordance of stated and revealed preferences for incentives in vaccination uptake.

## Methods

### Design and Setting

Housing for Health (HFH), a division of the Los Angeles County Department of Health Services, has been holding COVID-19 vaccination clinics for unhoused people since February 2021. HFH serves both sheltered and unsheltered individuals; 72% of unhoused people in Los Angeles County are unsheltered, meaning they live on the street, in makeshift shelters, in tents, or in vehicles [21]. In order to reach both sheltered and unsheltered individuals, vaccination clinics are held at homeless shelters, homeless services centers, encampments, parks, and recycling centers. During these events, food, water, hygiene products, harm reduction supplies, and clinical services such as wound care and blood pressure checks are provided alongside vaccination and/or vaccine education. Clients could access any other resource or service regardless of whether they got vaccinated. On average, HFH holds 40 of these events at locations with under-vaccinated populations across Los Angeles County each week.

For this analysis, HFH staff conducted a rapid, field-based, face-to-face, anonymous survey of unhoused people in Los Angeles County. Individuals were approached during HFH-organized COVID-19 vaccination clinics from May through November 2021. Staff canvassed the local area by car and on foot to reach a broader sample of unhoused people who could potentially get vaccinated at the event. An attempt was made to speak with every individual who appeared to be unhoused (e.g., shelter residents, at an encampment, accessing homeless services) in the immediate vicinity. Outreach workers offered snacks, water, and harm reduction supplies before asking about vaccination.

The serial cross-sectional survey was conducted in three iterative rounds in order to inform the development of programs that address the needs of unhoused people during the rapidly changing COVID-19 pandemic. In order to prevent losing trust and rapport with clients, the survey was limited to two questions and neither identifying nor demographic information were collected. Rounds 1 and 2 included only unsheltered participants, and round 3 included both unsheltered and sheltered participants. Due to the anonymous nature of the survey, some participants may have been included in more than one round or more than once in one round.

### Measures

COVID-19 vaccination status and interest were assessed during all three rounds. An additional unique question based on current programmatic needs was included in each round. Round 1 (May 2021) assessed vaccine brand preference; round 2 (June–August 2021) assessed interest in an incentive for vaccination; round 3 (September–November 2021) assessed reasons for vaccine readiness and hesitancy and evaluated implementation of an incentive program.

COVID-19 vaccination status was assessed via self-report. Unvaccinated participants were asked if they wanted to get vaccinated and answered yes, no, or not yet. For each survey round, simultaneous 95% confidence intervals for multinomial proportions were calculated using the R package MultinomialCI [22].

During round 1, those who answered yes were asked if they preferred the two-dose Moderna vaccine, the single-dose Johnson & Johnson vaccine, or either (at the time HFH did not offer the Pfizer vaccine). A chi-squared test for equality of proportions was used to test for a difference in brand preference.

During round 2, those who indicated they were not yet ready or did not want to get vaccinated were asked if being offered a $50 gift card would change their mind about getting vaccinated; participants ranked their responses on a 5-point Likert scale: 1 definitely not, 2 probably not, 3 possibly, 4 very possibly, and 5 definitely. The value of $50 was recommended by local health authorities to align with the state of California’s “Vax for the Win” incentive program, which offered the general public a $50 gift card to get vaccinated [23]. Aligning with the state program promoted parity for unhoused people and discouraged individuals from searching for the highest incentive.

Ability of an incentive to change one’s mind about vaccination was compared among those who did not want to get vaccinated and those who were not yet ready using a chi-squared test. Whether or not an incentive would change one’s mind about getting vaccinated was categorized as yes (very possibly, definitely), maybe (possibly), or no (probably not, definitely not).

During round 3, participants who indicated they were ready to get vaccinated were asked if they had actually been vaccinated that day as well as provided their primary reasons for choosing to get vaccinated now (vaccine readiness). Participants who indicated they were not yet ready or did not want to get vaccinated were asked to provide their primary reasons for choosing not to get vaccinated (vaccine hesitancy). Based on this open-ended conversation, field staff chose up to three reasons from a list of 14 reasons for vaccine readiness and 17 reasons for vaccine hesitancy (Table 3 and Table 4). The list of reasons for vaccine readiness and hesitancy was developed from a listening session where HFH staff were asked to offer common reasons for vaccine readiness and hesitancy they encountered while doing outreach.

Reasons for vaccine readiness and hesitancy were compared for unsheltered and sheltered participants. Reasons for vaccine hesitancy were also compared for participants who indicated they were not yet ready to get vaccinated and participants who did not want to get vaccinated. All comparisons were calculated using a chi-squared test for large samples or a Fisher’s exact test for small samples (an expected cell size < 5). At this time, an incentive program offering $50 gift cards for vaccination was implemented; in order to evaluate acceptability of the program, being offered a gift card was included as a reason for vaccine readiness and feeling coerced/bribed by the gift card was included as a reason for vaccine hesitancy.

### Statistical Analysis

In order to reduce the false discovery rate, a *P* value of less than 0.01 was considered statistically significant for all analyses [24]. Analyses were conducted using R Version 4.1.0 [25]. The study was determined by the University of California, Los Angeles, IRB to be exempt from IRB oversight.

## Results

A total of 5177 individuals were approached, and 228 (4.4%) declined to participate. Of the 4949 participants, 2008 (40.6%) reported being already vaccinated, 1732 (35.0%) were ready to get vaccinated, 359 (7.3%) were not yet ready to get vaccinated, and 850 (17.2%) did not want to get vaccinated. The distribution of vaccination status and readiness at each survey round is presented in Table 1.

**Table 1:** COVID-19 vaccine readiness among unhoused people, May–November 2021 (*n* = 4949)

	Round 1		Round 2		Round 3			
	Unsheltered (*n* = 598)		Unsheltered (*n* = 654)		Unsheltered (*n* = 2384)		Sheltered (*n* = 1313)	
	*n* (%)	95% CI	*n* (%)	95% CI	*n* (%)	95% CI	*n* (%)	95% CI
Already Vaccinated	214 (35.8)	(31.6–40.2)	240 (36.7)	(32.6–40.8)	1021 (42.8)	(40.7–45.0)	533 (40.6)	(37.7—43.5)
Yes	203 (33.9)	(29.8–38.3)	223 (34.1)	(30.0–38.2)	743 (31.2)	(29.0–33.4)	563 (42.9)	(40.0–45.8)
Not Yet	84 (14.0)	(9.9–18.4)	51 (7.8)	(3.7–11.9)	164 (6.9)	(4.7–9.1)	60 (4.6)	(1.7–7.5)
No	97 (16.2)	(12.0–20.6)	140 (21.4)	(17.3–25.5)	456 (19.1)	(17.0–21.3)	157 (12.0)	(9.1–14.9)

Of the 203 unsheltered participants who were ready to get vaccinated during round 1, 63 (31.0%) preferred the two-dose Moderna COVID-19 vaccine, 72 (35.5%) preferred the single-dose Johnson & Johnson COVID-19 vaccine, and 68 (33.5%) did not have a preference. There was no statistically significant difference in brand preference (*p* = 0.74).

During round 2 of the study, among 191 unsheltered participants who answered no or not yet when asked if they wanted to get vaccinated, 36 (24.2%) indicated that an incentive would change their mind, 29 (19.5%) indicated that it might, 84 (56.4%) indicated that it would not, and 42 declined to answer. Among the 44 participants who were not yet ready to get vaccinated, 37 (84.1%) of said that an incentive would definitely or possibly change their mind. Conversely, 77 (73.3%) of the 105 participants who did not want to get vaccinated said that an incentive would not change their mind. There was a statistically significant difference in incentive interest among those who were not yet ready to get vaccinated and those who did not want to get vaccinated (*p* < 0.01) (Table 2).

**Table 2:** Incentive interest among unvaccinated unsheltered unhoused people, June–August 2021 (*n* = 149)

	Do you want to get vaccinated?			
	Total (*n* = 149)*	Not Yet (*n* = 44)	No (*n* = 105)	*P value*
Would a $50 gift card change your mind about getting vaccinated?				
Yes	36 (24.2)	19 (43.2)	17 (16.2)	< 0.01
Maybe	29 (19.5)	18 (40.9)	11 (10.5)	
No	84 (56.4)	7 (15.9)	77 (73.3)	

^*^42 of 191 participants answering not yet or no declined to answer gift card interest question

During round 3, 97.4% of the 1306 individuals who indicated they were ready to get vaccinated were actually vaccinated that day. The percentage of individuals who were actually vaccinated that day did not differ for unsheltered (98.1%) and sheltered (96.4%) participants (*p* = 0.09). In addition, 731 individuals (56.0%) cited the $50 gift card as a primary reason for why they decided to get vaccinated now. Other common reasons for vaccine readiness were to protect others (24.0%) and because outreach staff recommended it (15.6%). Unsheltered participants were more likely to cite the $50 gift card (67.2% vs 41.2%, *p* < 0.01) and recommendation by HFH staff (20.9% vs. 8.7%, *p* < 0.01); sheltered participants were more likely to cite a desire to protect others (30.0% vs. 19.5%, *p* < 0.01), concern about the Delta variant (17.8% vs. 10.1%, *p* < 0.01), and perceived mandates for housing and/or job (28.4% vs. 4.0%, *p* < 0.01; 20.8% vs. 3.1%, *p* < 0.01). Additional comparisons are presented in Table 3.

**Table 3:** Reasons for vaccine readiness among unhoused people, September–November 2021 (*n* = 1306)

	Total (*n* = 1306)	Unsheltered (*n* = 743)	Sheltered (*n* = 563)	*P* value
Incentive ($50 gift card)	731 (56)	499 (67.2)	232 (41.2)	< 0.01
Protect others (community, friends, family)	314 (24)	145 (19.5)	169 (30)	< 0.01
HFH staff recommended it	204 (15.6)	155 (20.9)	49 (8.7)	< 0.01
Perceived required/mandated for housing	190 (14.5)	30 (4)	160 (28.4)	< 0.01
Delta variant/rising cases/more contagious	175 (13.4)	75 (10.1)	100 (17.8)	< 0.01
Required/mandated for their job	140 (10.7)	23 (3.1)	117 (20.8)	< 0.01
Know others who got safely vaccinated	79 (6)	34 (4.6)	45 (8)	0.01
Said 'why not' or had nothing else to do	73 (5.6)	43 (5.8)	30 (5.3)	0.81
It was their first chance to get vaccinated	53 (4.1)	25 (3.4)	28 (5)	0.19
Required for restaurant, sporting event, etc	36 (2.8)	17 (2.3)	19 (3.4)	0.31
Vaccine is fully FDA-approved	23 (1.8)	4 (0.5)	19 (3.4)	< 0.01
Kids going back to school	23 (1.8)	5 (0.7)	18 (3.2)	< 0.01
Tired of being told to get vaccinated	20 (1.5)	8 (1.1)	12 (2.1)	0.17

Of the 837 participants who were not yet ready or did not want to get vaccinated during round 3, 240 (28.7%) declined to answer why they were hesitant about vaccination, and 175 (20.9%) said that getting vaccinated was not a top priority or that they had more pressing needs. Other common reasons included not being concerned about or afraid of getting COVID-19 (15.5%), concerns about vaccine safety (14.6%), and concerns about side effects (9.4%). Unsheltered participants were more likely to decline to answer (32.6% vs. 17.5%, *p* < 0.01) and sheltered participants were more likely to have concerns about side effects (12.9% vs. 8.2%, *p* < 0.01). Those who did not want to get vaccinated were more likely to decline to answer (31.8% vs. 17.5%, *p* < 0.01) and those who were not yet ready to get vaccinated were more likely to say vaccination was not a top priority (29.0% vs. 17.9%, *p* < 0.01) and have concerns about side effects (15.2% vs. 7.3%, *p* < 0.01). Additional comparisons are presented in Table 4.

**Table 4:** Reasons for vaccine hesitancy among unhoused people, September–November 2021 (*n* = 837)

		Shelter status			Vaccine readiness			
	Total (*n* = 837)	Unsheltered (*n* = 620)	Sheltered (*n* = 217)	*P* value	Not yet (*n* = 224)	No (*n* = 613)		*P* value
Decline to answer	240 (28.7)	202 (32.6)	38 (17.5)	< 0.01	45 (20.1)	195 (31.8)		< 0.01
Not a top priority for them/have more pressing needs	175 (20.9)	124 (20)	51 (23.5)	0.32	65 (29)	110 (17.9)		< 0.01
Not concerned about or afraid of getting COVID-19	130 (15.5)	104 (16.8)	26 (12)	0.12	30 (13.4)	100 (16.3)		0.36
Vaccine is not safe/may cause serious health complications	122 (14.6)	81 (13.1)	41 (18.9)	0.05	28 (12.5)	94 (15.3)		0.36
Worried about having a bad reaction/side effects	79 (9.4)	51 (8.2)	28 (12.9)	< 0.01	34 (15.2)	45 (7.3)	< 0.01	
Distrust healthcare system due to historic/current racism	63 (7.5)	50 (8.1)	13 (6)	0.40	18 (8)	45 (7.3)	0.85	
Concerned about vaccine development process	51 (6.1)	33 (5.3)	18 (8.3)	0.16	18 (8)	33 (5.4)	0.21	
Not at risk or at low risk of getting COVID-19	50 (6)	34 (5.5)	16 (7.4)	0.40	12 (5.4)	38 (6.2)	0.77	
Vaccine will not work/is ineffective	45 (5.4)	30 (4.8)	15 (6.9)	0.32	8 (3.6)	37 (6)	0.22	
Know someone who had a bad experience/side effects	40 (4.8)	24 (3.9)	16 (7.4)	0.06	14 (6.2)	26 (4.2)	0.31	
Vaccine may infect them with COVID-19	39 (4.7)	26 (4.2)	13 (6)	0.37	13 (5.8)	26 (4.2)	0.44	
Against their religion	38 (4.5)	32 (5.2)	6 (2.8)	0.20	2 (0.9)	36 (5.9)	< 0.01	
Vaccine is too new, not all are FDA approved	25 (3)	16 (2.6)	9 (4.1)	0.35	14 (6.2)	11 (1.8)	< 0.01	
Vaccine contains a microchip/tracking device	23 (2.7)	22 (3.5)	1 (0.5)	0.01	7 (3.1)	16 (2.6)	0.64	
Need more info / need to do own research	15 (1.8)	12 (1.9)	3 (1.4)	0.77	10 (4.5)	5 (0.8)	< 0.01	
Felt coerced/bribed by incentive (gift card)	14 (1.7)	13 (2.1)	1 (0.5)	0.13	2 (0.9)	12 (2)	0.37	
Vaccination will lower vulnerability score	3 (0.4)	2 (0.3)	1 (0.5)	0.99	1 (0.4)	2 (0.3)	0.99	

## Discussion

This study identified reasons for vaccine readiness and hesitancy among unhoused people in Los Angeles County. More sheltered individuals reported being already vaccinated or ready to get vaccinated than unsheltered individuals. This may be explained by an increased awareness of COVID-19 in sheltered settings, where outbreaks, quarantine, isolation, and required risk mitigation methods such as masking and social distancing are common [26, 27]. In addition, those living in unsheltered settings may feel that living outdoors reduces their risk of acquiring COVID-19 compared to living in a congregate setting despite having limited access to essential needs such as food, water, and hygiene [28].

Round 1 found that 66.5% of participants preferred a specific vaccine brand, but no one brand was widely favored. In the 6 months following round 1, stated preference matched revealed action for vaccination: 47.5% of first doses administered by HFH were single dose Johnson & Johnson vaccines, and 52.5% were two dose mRNA vaccines. This reinforces the importance of multiple options and suggests the Johnson & Johnson pause may not have done lasting damage to vaccine confidence in this population.

Round 2 analyses demonstrated that financial incentives could be a useful tool for increasing COVID-19 vaccine uptake among unhoused people in Los Angeles County. In particular, the study results indicated that this intervention might be most effective among those who are not yet ready to get vaccinated; 84.1% of this group indicated that a $50 gift card would definitely or maybe change their mind about vaccination compared to 26.7% of those who did not want to get vaccinated.

Despite these differences, the high levels of vaccination interest among participants during round 2 suggested that financial incentives could impact COVID-19 vaccine uptake in this population in a meaningful way. Among the 63.3% of participants who were unvaccinated, 66.2% said they were interested in getting vaccinated (53.9% were ready to get vaccinated immediately and 12.3% wanted to get vaccinated but were not yet ready).

Based on these results, HFH implemented a program to offer $50 gift cards for first doses during round 3 of the study; 56% of those who were ready to get vaccinated cited the incentive as a primary deciding factor, and nearly everyone who was ready to get vaccinated got vaccinated the same day they completed the survey. Additionally, only 1.7% of participants who did not want to get vaccinated reported feeling coerced or bribed by the incentive. These results support other findings that suggest financial incentives are acceptable to the public (including unhoused people) and could improve vaccine uptake, particularly among those who are ambivalent about vaccination. [9, 29].

This study also identified key reasons for vaccine readiness and hesitancy among unhoused people in Los Angeles County. The finding that unsheltered people were more likely to endorse the gift card incentive and HFH staff recommendations as reasons to get vaccinated reinforced the strategy of personal engagement and material assistance for people who may be least connected to social services. Even though shelter residents were less likely than unsheltered people to endorse the gift card incentive, it was still the most common reason for vaccine readiness in this group and suggests that financial incentives are also important in sheltered settings. The difference in factors endorsed by shelter residents compared to unsheltered people may reflect the shelter environment: for example, living in a congregate indoor setting likely provides more opportunities to observe others experiencing side effects. The motivation to protect others, a top reason for both groups but significantly more common among shelter residents, illustrates how concern for community may be a powerful motivator to take personal health action. This appears to be especially salient for people living in closer indoor quarters. Based on these findings, HFH staff were able to develop evidence-based education and outreach materials including flyers and games that addressed the specific concerns of unhoused people.

Individuals who did not want to get vaccinated were less willing to discuss vaccination with outreach staff and were less open to financial incentives. This finding suggests that different strategies and additional efforts will be required to promote vaccination in this group. Among those who did not want to get vaccinated but were willing to discuss vaccination, the top reason for not wanting to get vaccinated was that it was not a top priority due to more pressing needs; providing clients with food, hygiene, referrals to housing, and medical and mental health care may assist them in meeting their basic needs and allow them to prioritize COVID-19 prevention [30, 31].

Because this study used a convenience sample and did not measure demographic characteristics of participants, the generalizability of these results may be limited beyond HFH clients, and relevant differences in attitudes toward vaccination across demographic groups could not be captured. Unhoused people in Los Angeles are certainly not homogenous, and demographic differences in vaccine uptake are key metrics locally and more broadly [10]. We briefly piloted including demographic questions (age, gender identity, race, and ethnicity) after the vaccine readiness question, but clients were often unwilling to answer these questions. Research staff and outreach staff overwhelmingly felt that asking these questions was damaging their rapport with clients and counterproductive to future outreach. For these same reasons, we did not directly ask people about their housing status as part of the survey. It is possible that some people who appeared to be living in a vehicle, who were spending time at an encampment, who were recycling cans at a recycling center, or who were accessing public services such as food lines or showers did have permanent housing. However, given these individuals were part of the social and geographic networks of unhoused people, we took the view that their vaccination status was directly relevant to the community HFH serves.

While individuals may have been surveyed more than once, HFH clinics often target new locations with under-vaccinated populations, so the number of individuals included more than once is likely negligible. Targeting under-vaccinated populations also means that this study’s estimates of vaccination coverage likely underestimate coverage in the general population of unhoused people in Los Angeles County. Also, individuals who declined to discuss vaccination during round 1 were recorded as not wanting to get vaccinated; this may have resulted in misclassification of vaccine readiness for some individuals. In addition, “already vaccinated” status may be subject to social desirability bias because it was determined via self-report; some participants who did not want to get vaccinated could have reported being vaccinated in order to quickly end the conversation.

However, the ability to match stated intention to get vaccinated to actual behavior of getting vaccinated, and the high level of agreement in round 3 is a major strength of this study. In addition, the self-reported “already vaccinated” rates were consistent with vaccination coverage estimates reported at that time by the Department of Public Health. Moreover, this study demonstrated the utility of an iterative, field-based assessment for program implementation and evaluation during the rapidly changing COVID-19 pandemic.

Although COVID-19 vaccine uptake has consistently been lower among unhoused people in Los Angeles County compared to the general population, the findings of this study suggest that interest is high. Crucially, vaccine interest may change over time, and some declinations are better understood as not yet rather than never. Longitudinal assessments of the same persons would help evaluate how interest may change as the pandemic unfolds and different strains enter into circulation. Ongoing evaluation of the impact of financial incentives as well as additional research about how to engage unhoused people who are not interested in vaccination is needed to further improve vaccine readiness in this vulnerable population.
